# Ultra-Wideband Transformer Feedback Monolithic Microwave Integrated Circuit Power Amplifier Design on 0.25 μm GaN Process

**DOI:** 10.3390/mi15040546

**Published:** 2024-04-18

**Authors:** Jialin Luo, Yihui Fan, Jing Wan, Xuming Sun, Xiaoxin Liang

**Affiliations:** 1 Institute of Microelectronics of the Chinese Academy of Sciences, Beijing 100029, China; 2 University of Chinese Academy of Sciences, Beijing 101408, China; 3 Beijing Key Laboratory of New Generation Communication RF Technology, Beijing 100020, China

**Keywords:** MMIC, TFB, wideband matching, transmission line theory

## Abstract

This paper presents an ultra-wideband transformer feedback (TFB) monolithic microwave integrated circuit (MMIC) power amplifier (PA) developed using a 0.25 μm gallium nitride (GaN) process. To broaden the bandwidth, a drain-to-gate TFB technique is employed in this PA design, achieving a 117% relative −3 dB bandwidth, extending from 5.4 GHz to 20.3 GHz. At a 28 V supply, the designed PA circuit achieves an output power of 25.5 dBm and a 14 dB small-signal gain in the frequency range of 6 to 19 GHz. Within the 6 to 19 GHz frequency range, the small-signal gain exhibits a flatness of less than 0.78 dB. The PA chip occupies an area of 1.571 mm^2^. This work is the first to design a power amplifier with on-chip transformer feedback in a compound semiconductor MMIC process, and it enables the use of the widest bandwidth power amplifier on-chip transformer matching network.

## 1. Introduction

To cope with the growing volume of data transmission and the requirement for transmission rate in the 5G era, broadband systems are receiving increasing attention and have become a trend in the development of communication systems. In fields such as national defense, frequency hopping systems required for electronic countermeasures or high-resolution radar surveillance systems also require a wide bandwidth operating space, which puts high demands on the actual RF microwave systems. According to Shannon’s theorem, the expansion of channel capacity can be achieved by increasing the bandwidth with a certain signal-to-noise ratio, which is highly instructive for modern communication systems.

As a key component of RF systems, power amplifiers in the form of monolithic microwave integrated circuits (MMICs) are highly valued by designers because of their small size, light weight, and high reliability, and their high level of integration plays an important role in a variety of electronic system fields. There exist numerous design approaches for wideband PAs, encompassing strategies such as reactive matching, transformer matching [1], and distributed topology, among others. For wideband systems, particularly when the relative bandwidth exceeds 40%, feedback networks are typically utilized in the design of amplifier models. As the bandwidth widens, the optimal impedance of the transistor varies sharply, and the gain roll-off characteristic becomes more pronounced. These factors make broadband matching more difficult. The reason for this phenomenon is partly due to the larger volume and more complex parasitic coupling effects of GaN HEMT transistors, especially the parasitic capacitance (Cgd) at the gate and drain terminals of transistors that cannot be ignored. To address these challenges, traditional wideband PA designs often resort to methods such as low-Q reactive impedance matching, balanced structures [2,3], or resistive shunt feedback [4,5]. However, these solutions tend to be less efficient and consume larger chip areas. As a better alternative, some research has begun to use on-chip transformer feedback in broadband amplifier designs to build feedback matching networks [6,7,8].

Transformer feedback (TFB) has found extensive applications in high-frequency circuits [1,9,10,11,12,13,14,15,16]. Within a single-stage amplifier, the drain-to-source and drain-to-gate TFB configurations serve to counterbalance the device gate–drain capacitance [9,10]. This method has been integrated into broadband amplifiers to boost bandwidth [15,16] and diminish noise levels [17,18]. Thus, the use of TFB technology has great application prospects for achieving good broadband performance.

In this paper, the design procedure for a 6–19 GHz transformer feedback (TFB) GaN power amplifier with a gain flatness of 0.78 dB is described. The TFB method in detail based on the distributed transformer modeling theory is proposed in this PA to achieve an ultra-wideband performance. The remainder of this paper is organized as follows: Section 2 will discuss different transformer design process parameters and the influence on the transformer performance. Section 3 will focus on the analysis of the TFB technique. Section 4 will cover the circuit design and the related measurement results, respectively. The conclusion of this work will be discussed in the final section, Section 5.

## 2. Transformer Design Process Comparison

For over half a century, under the guiding principle of Moore’s law, the silicon-based CMOS integrated circuit has stood as the cornerstone of the information society. At present, silicon semiconductor scaling has progressed to the nanometer level, encountering technical and performance limitations stemming from the inherent constraints of silicon’s performance characteristics. In pursuit of high-performance and ultra-wideband transceivers operating in the microwave spectrum, research has delved into III-V compound semiconductor technologies such as gallium arsenide (GaAs), indium phosphide (InP), aluminum nitride (AlN), and gallium nitride (GaN). These materials offer promising solutions for solid-state power amplifiers that are high-powered, cost-effective, and reliable. III-V compound semiconductor technology boasts superior transistor characteristics in high-frequency applications, while the semi-insulating substrate facilitates the integration of high-quality passive components with minimal parasitic elements. Table 1 presents a comparative overview of the properties of various semiconductor materials.

**Figure 1:**
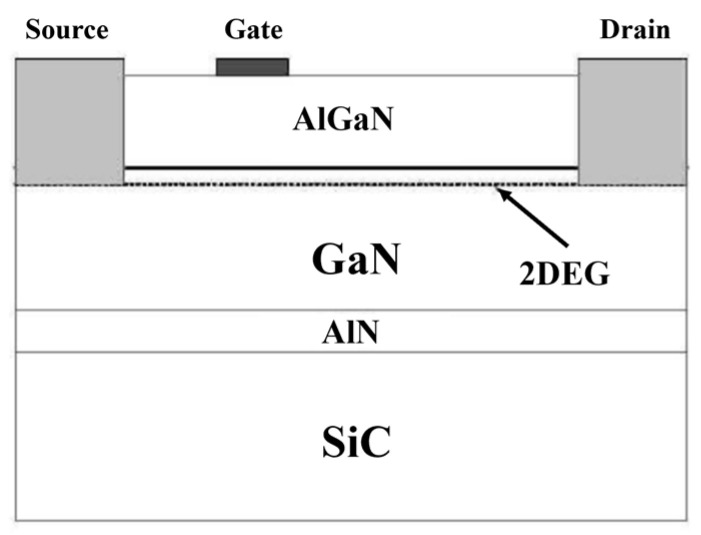
Typical AlGaN/GaN HEMT structure.

Compared with other materials, GaN materials have various characteristics such as a high breakdown voltage, high electron saturation rate, and high thermal conductivity, making devices made from these materials very suitable for RF applications. As shown in Figure 1, the creation of two-dimensional electron gas (2-DEG) channels in GaN HEMTs is achieved through AlGaN/GaN heterojunctions, where a wide bandgap semiconductor (AlGaN) interfaces with a narrow bandgap semiconductor (GaN). By establishing a 2-DEG channel within an undoped pure GaN layer, the impact of scattering by dopants is minimized, enabling the attainment of high electron mobility. The drain and source electrodes are both Ohmic contacts connected to the two-dimensional electron gas, with gate voltage controlling the two-dimensional electron gas beneath the gate Schottky junction to modulate the channel current. The reason for the gate electrode being closer to the source electrode in the structure is that in AlGaN/GaN HEMTs, if breakdown occurs during operation, the breakdown typically happens on the side of the gate closer to the drain electrode. This asymmetric structure can enhance the breakdown voltage, allowing the device to operate at higher drain-source voltages. GaN power devices operating at high current densities and voltages generate significant heat, leading to self-heating effects that can diminish the drain current, limit output power, and shorten device lifespan. Efficient heat dissipation is crucial, and one effective method involves releasing heat to the substrate. Silicon carbide (SiC) is a commonly used substrate for epitaxial growth in GaN HEMT technology due to its high thermal conductivity and low thermal resistance. The GaN MMIC process is mainly divided into two parts: front-side process and back-side process. The front-side process mainly produces GaN HEMT active devices and passive components such as resistors, MIM capacitors, microstrip lines, etc.; the back-side process includes wafer thinning, back-side via holes, and back-side metal deposition, which is used to form the ground plane of microstrip lines.

The performance of a fabricated inductor or transformer is determined by the IC technology process. This process involves several parameters related to the transformer, including the thickness of the top metal layer, metal conductance, minimum metal space, the distance of the metal layers from the substrate, the dielectric constant of the substrate, and so on. Due to parasitic and various loss mechanisms, the impedance transformation ratio is highly dependent on frequency and size and can deviate from its ideal value of 1, even in the case of a 1:1 transformer [19]. While some of these parameters are predefined in the technology process, they can still serve as a reference for transformer design.

Table 2 provides a comparison of major RF technology process parameters. The advanced CMOS process with a smaller feature size has more metal layers and a thicker top metal layer. For instance, the top metal conductance significantly improves to 5.816 × 10^7^ S/m in the TSMC 0.13 µm CMOS process. The number of metal layers increases from 5 to 8 when transitioning from the TSMC 0.25 µm to the 0.13 µm CMOS process. In contrast, III-V compound processes like GaAs and GaN, also listed in this table, have only two metal layers, but they exhibit larger metal conductance and layer spacing. In the 0.25 µm GaN process, the top metal thickness reaches 4 µm, which is the thickest among the processes listed. Designing an on-chip transformer using a thicker metal layer can effectively increase the coupling coefficient and reduce loss. Additionally, it helps to increase the transformer’s self-resonant frequency (f_srf_), which is highly beneficial in practical design.

Therefore, in comparison to CMOS technology, the GaN process features a thicker top metal layer and larger metal conductance. However, its metal layers are closer to the substrate, leading to a higher degree of substrate loss. The GaN process also has a comparatively larger minimum metal layer space, with 6 µm for the MET2 layer. This characteristic may limit the coupling coefficient of a designed coplanar transformer to some extent.

## 3. Analysis of Transformer Feedback Technique

**Figure 2:**
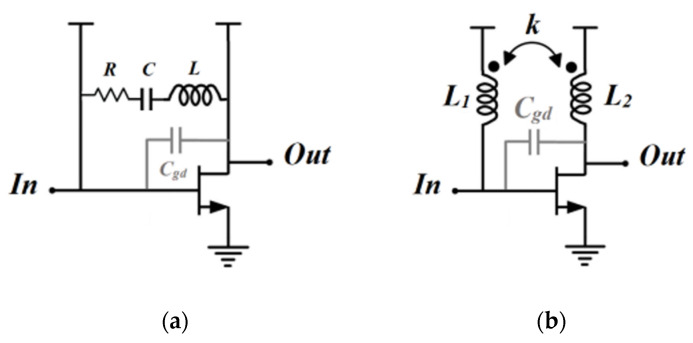
(**a**) The traditional RLC feedback common source (CS) amplifier; (**b**) the transformer feedback CS amplifier.

In GaN transistors, the parasitic capacitance (Cgd) and its associated Miller effect pose significant challenges for wideband matching. Moreover, the parasitic negative feedback caused by Cgd between the gate and drain limits the power gain and reverse isolation, potentially leading to instability [20,21]. To counteract these issues, the gate–drain feedback mechanism is used to neutralize Cgd, broaden the maximum available gain/power gain frequency response, and ensure stability. Instead of the conventional RLC reactive feedback network (as seen in Figure 2a), TFB offers an alternative approach for wideband PA design. Figure 2b illustrates the schematic design of a gate–drain TFB amplifier. Here, L_1_ and L_2_ represent the inductance at the gate and drain biasing circuits, respectively, while k denotes the coupling coefficient between the primary and secondary windings. The TFB technique operates on the principle that the secondary inductance (L_2_) senses the output current, and a proportion of this is fed back to the PA input through the primary inductance (L_1_) [22]. This technique does sacrifice a portion of the transistor’s power gain, but it results in a flattened frequency response and expanded bandwidth. Furthermore, the use of a transformer feedback network can adjust the amplifier port impedance, which can simplify the matching circuit design to a certain degree.

**Figure 3:**
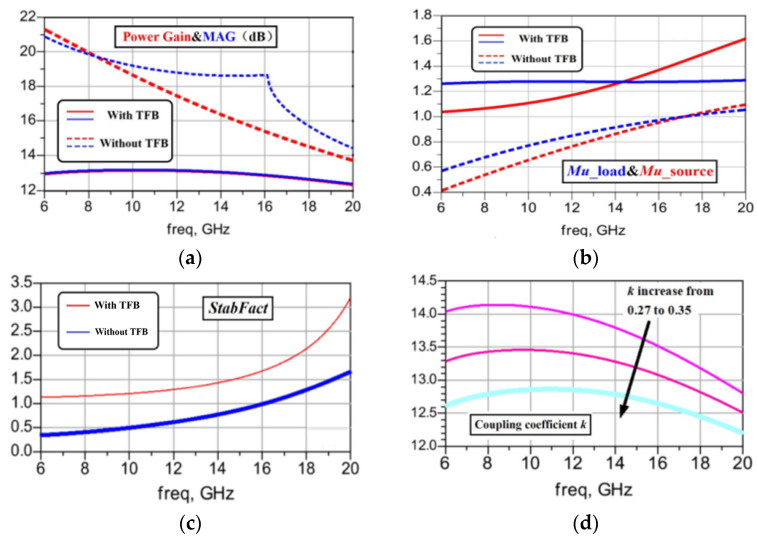
TFB effect to CS amplifier: (**a**) MAG/power gain; (**b**) stability factor Mu; (**c**) stability factor StabFact; (**d**) MAG vs. coupling coefficient.

As illustrated in Figure 2b, the TFB amplifier can be configured with an L1 inductance of 0.9 nH, an L2 inductance of 0.5 nH, and a coupling coefficient of 0.32. The simulation results, presented in Figure 3a, reveal that by applying the TFB technique, the amplifier’s maximum available gain (MAG) and power gain extend to an ultra-wide range. The amplifier demonstrates an impressively flat frequency response, exhibiting a deviation of less than 1 dB across the range of 6 to 20 GHz. Evidently, the feedback network compensates for the transistor gain roll-off performance, simplifying the wideband matching circuit design as it does not require intentional adjustment of the insertion loss gain roll-off based on transistor performance trends. However, the amplifier’s gain performance is compromised. Without the TFB network, the amplifier’s MAG and power gain are 18.798 dB and 17.31 dB @12 GHz, respectively. After incorporating the feedback, these values drop to 13.28 dB and 13.35 dB @12 GHz, respectively.

In Figure 3b,c, stability factors such as Mu and StabFact are shown to be below one. However, after the implementation of the TFB network, these stability factors of the amplifier improve to values above one. The coupling coefficient, represented by k, is utilized to adjust the depth of feedback. As depicted in Figure 3d, when k increases from 0.27 to 0.35, there is a steady reduction in the amplifier’s maximum available gain (MAG) from 13.97 dB to 12.85 dB @12 GHz. Additionally, in the lower frequency band, the MAG curve exhibits a more rapid decline than in the high-frequency band, as shown in Figure 3a.

**Figure 4:**
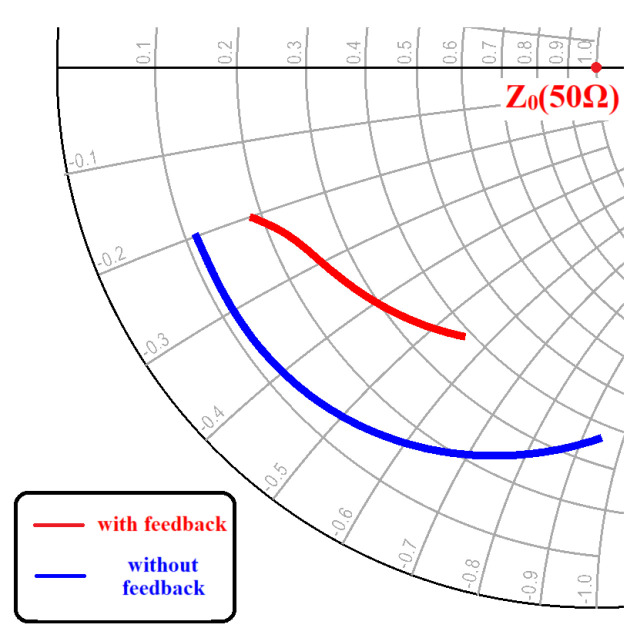
The CS amplifier input return loss S11 trace on the Smith chart.

Furthermore, the use of on-chip transformer feedback aids in adjusting the amplifier’s input impedance. If the amplifier’s gate impedance is brought closer to the 50 Ω impedance range via the feedback network, it can decrease the impedance transformation ratio of the input matching circuit, thus simplifying the design of the input matching circuit. The feedback network’s inductance L1/L2 and coupling coefficient k can be adjusted to modify the gain and input impedance of the on-chip transformer feedback amplifier. In this particular design, the on-chip transformer feedback network is primarily used to broaden the amplifier’s bandwidth and offset the frequency-dependent roll-off characteristics of the transistor. As shown in the Smith chart in Figure 4, the blue line represents the S11 of the CG amplifier without the on-chip transformer feedback network, while the red line represents the S11 of the amplifier with the on-chip transformer feedback. It is clear that the addition of the on-chip transformer feedback network brings the amplifier’s input impedance closer to the center point, Z0 (50 Ω).

**Figure 5:**
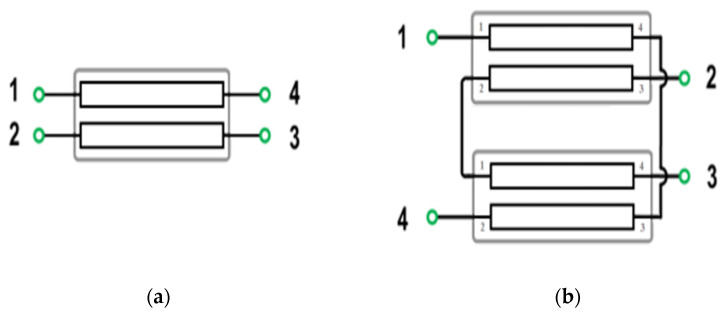
(**a**) The coupling TL section view; (**b**) the transformer distribute model based on coupling TL.

For a more in-depth analysis of the TFB mechanism in broadband PA design, we also examine a four-port transformer model in this paper. The traditional symmetric 2π transformer model [23] is complex, with numerous parameters that need to be fitted. For the sake of simplicity, we opt for a transmission line (TL) theory-based distributed transformer model for our analysis, as depicted in Figure 5.

The inductance at the biasing circuit can be realized by TL. The coupling effect between the primary and secondary windings of the transformer can be modeled as the coupling TL [24,25,26], which captures both magnetic (inductive) and electric (capacitive) couplings between the two windings [27]. By employing this method, a four-port coupling TL Y-matrix can be expressed as follows [28]:

(1) $$Y=\left[\begin{array}{l} -jY_{p}\cot\theta \;\;-jY_{m}\cot\theta \;\;\;\;\;jY_{m}\cot\theta \;\;\;\;\;jY_{p}\cot\theta \\ -jY_{m}\cot\theta \;\;-jY_{p}\cot\theta \;\;\;\;\;jY_{p}\csc\theta \;\;\;\;\;jY_{m}\csc\theta \\ \;\;jY_{m}\csc\theta \;\;\;\;\;jY_{p}\csc\theta \;\;-jY_{p}\cot\theta \;\;-jY_{m}\cot\theta \\ \;\;jY_{p}\csc\theta \;\;\;\;\;jY_{m}\csc\theta \;\;-jY_{m}\cot\theta \;\;-jY_{p}\cot\theta \end{array}\right]$$

where

(2) $$Y_{p}=\frac{Y_{0o}+Y_{0e}}{2}$$

(3) $$Y_{m}=\frac{Y_{0o}-Y_{0e}}{2}$$

(4) $$Y_{0e}=\frac{1}{Z_{0e}}$$

(5) $$Y_{0o}=\frac{1}{Z_{0o}}$$

The coupling coefficient, denoted as *k*, can be expressed as follows:

(6) $$k=\frac{Y_{0o}-Y_{0e}}{Y_{0o}+Y_{0e}}$$

*Z*_0*e*_/*Z*_0*o*_ represent TL even/odd characteristic impedance, and *θ* denotes the electrical length of the transmission line. The transformer can be conceptualized as two pairs of coupled transmission lines (TLs), as depicted in Figure 5b, which makes it a four-port device. The elements of its Y-parameter matrix can be expressed as follows:

(7) $$Y_{11}^{*}=Y_{44}^{*}=-Y_{11}+Y_{12}\cdot \frac{Y_{21}Y_{44}-Y_{41}Y_{24}}{2(Y_{22}Y_{44}-Y_{24}Y_{42})}+Y_{14}\cdot \frac{Y_{21}Y_{42}-Y_{41}Y_{22}}{2(Y_{24}Y_{42}-Y_{44}Y_{12})}$$

(8) $$Y_{12}^{*}=Y_{21}^{*}=Y_{34}^{*}=Y_{43}^{*}=-Y_{13}+Y_{32}\cdot \frac{Y_{21}Y_{44}-Y_{41}Y_{24}}{2(Y_{22}Y_{44}-Y_{42}Y_{24})}+Y_{34}\cdot \frac{Y_{21}Y_{42}-Y_{41}Y_{22}}{2(Y_{24}Y_{42}-Y_{44}Y_{22})}$$

(9) $$Y_{23}^{*}=Y_{32}^{*}=Y_{34}\cdot \frac{Y_{23}Y_{42}-Y_{43}Y_{22}}{2(Y_{22}Y_{44}-Y_{24}Y_{42})}+Y_{32}\cdot \frac{Y_{23}Y_{24}-Y_{43}Y_{24}}{2(Y_{22}Y_{44}-Y_{42}Y_{24})}$$

Figure 2b presents a simplified schematic of the transformer feedback (TFB) amplifier stage. If we assume that the transistor’s admittance is a 2 × 2 Y-parameter matrix, we can add the shunt TFB network’s Y-matrix to it. Consequently, the open-loop transformer and TFB loop can be expressed as follows [29]:

(10) $${\left[Y\right]}_{tol}={\left[Y\right]}_{transistor}+{\left[Y\right]}_{f}=\left[\begin{array}{l} y_{11}+y_{f}\;\;y_{12}-y_{f}\\ y_{21}-y_{f}\;\;y_{22}+y_{f} \end{array}\right]=\left[\begin{array}{l} y_{tf11}\;\;y_{tf12}\\ y_{tf21}\;\;y_{tf22} \end{array}\right]$$

where the feedback admittance, *y_f_*, corresponds to $Y_{34}^{*}$ in the equation.

The stability factor, referred to as StabFact, and the small-signal gain, denoted as S21, of the transformer feedback (TFB) amplifier can be derived as follows [30]:

(11) StabFact=2Re(ytf11)Re(ytf22)−Re(ytf12ytf21)ytf12ytf21

(12) $$S21=\frac{-2Y_{0}y_{tf21}}{(Y_{0}+y_{tf22})-y_{tf12}y_{tf21}}$$

**Figure 6:**
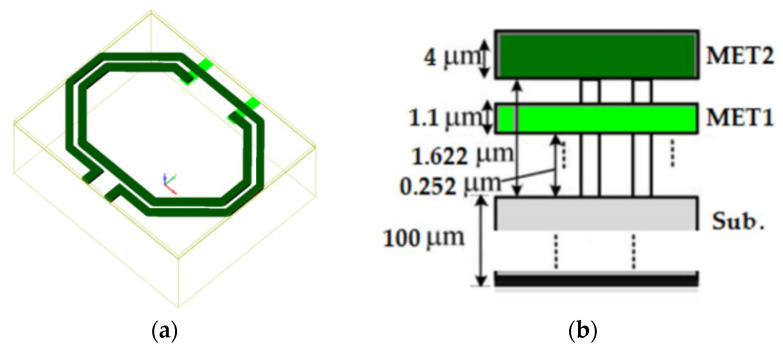
Transformer layout design: (**a**) the transformer design on 0.25 μm GaN process; (**b**) cross view of the 0.25 μm GaN process.

The transformer is designed using a 0.25 μm GaN process with a thick MET2 metal layer on top to achieve a higher Q factor and lower insertion loss. Figure 6a illustrates the transformer layout designed using the 0.25 μm GaN process, while Figure 6b presents a sectional view of the 0.25 μm GaN process. In the GaN process, two metal layers, MET1 and MET2, are available with thicknesses of 4 μm and 1.1 μm, respectively.

**Figure 7:**
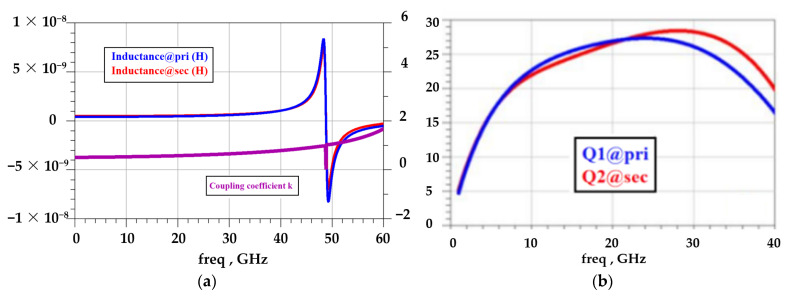
Transformer parameters extracted from EM simulation: (**a**) equivalent inductance at primary and secondary winding; (**b**) Q value of primary and secondary winding.

Due to the application of heteroepitaxial technology, the GaN metal layers are epitaxially grown on a 100 μm SiC substrate to improve the thermal characteristics of the device and reduce the junction temperature, as shown in Figure 6b. The transformer designed with this process exhibits a high Q value, as depicted in Figure 7b. The transformer inductance Q value reaches approximately 25 @25 GHz and remains above 20 across the entire band. Figure 7a illustrates the windings of the transformer. The primary winding has an equivalent inductance of 0.414 nH, while the secondary winding has an equivalent inductance of 0.498 nH @10 GHz. The coupling coefficient between the windings is 0.531. Both windings exhibit a self-resonant frequency (fsrf) of 48.8 GHz.

## 4. Circuit Design and Measurement

**Figure 8:**
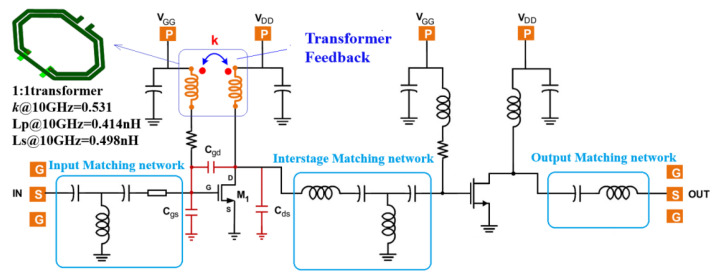
The schematic of proposed TFB power amplifier schematic design.

This section delves into the discussion of an ultra-wideband power amplifier leveraging the transformer feedback (TFB) technique. The circuit is designed and implemented using a 0.25 μm MMIC GaN process. The circuit schematic is depicted in Figure 8. Both stages of the amplifier structure are biased at Vg = −2 V, Vd = 28 V, class AB operation, with a quiescent current consumption of 130 mA. The sizes of the two-stage OSV HEMT transistors are 4 × 70 μm and 4 × 90 μm, respectively. The driving stage is configured to provide ample power and gain, with the target of enhancing the output power. The feedback network is engineered to compensate for the transistor gain ripple effect and to extend the bandwidth from 6 to 19 GHz. It also serves to address the instability induced by the parasitic capacitance Cgd. Employing a feedback network tends to limit transistor gain and output power. To achieve a larger output power, the second-stage amplifier abstains from using a feedback network. All the matching circuits are designed using the port impedance model (PIM) matching the circuit design method [31]. Based on the variation trend of the optimum impedance (Zopt) of the active device, the structure of the reactive matching circuit and the values of its components can be determined.

**Figure 9:**
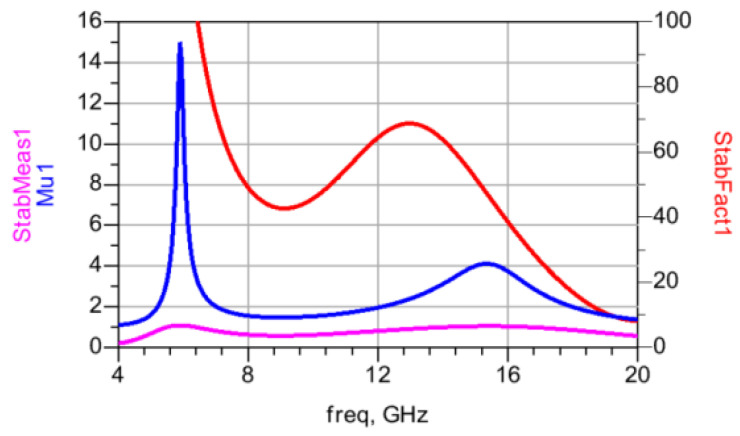
Stability factor StabFact, StabMeas, and Mu of the designed TFB PA.

The designed GaN MMIC process on-chip transformer and all passive matching structures were designed and verified using EM simulation in the ADS 2020 Momentum platform. To ensure the accuracy of the design results, the EM simulation models of the chip PAD and bonding lines are included in this design. The stability factors StabFact, StabMeas, and Mu are shown in Figure 9. The simulation results show that StabFact > 1 is in the extended frequency range with a minimum value of 6.58 @ 9.3 GHz, and Mu > 1 and StabMeas > 0. Therefore, the power amplifier of this design is unconditionally stable.

**Figure 10:**
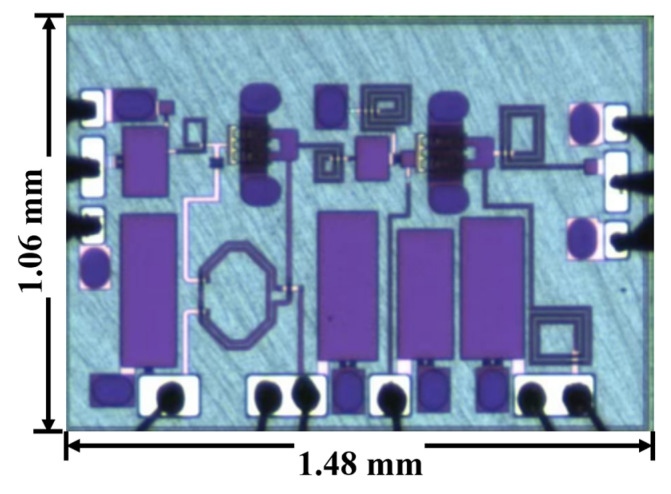
The fabricated chip microphotograph.

**Figure 11:**
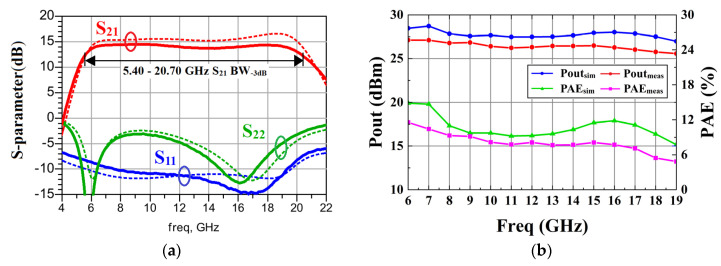
The measured and simulated results of TFB PA: (**a**) S-parameter; (**b**) large-signal performance.

Figure 10 shows the chip’s microphotograph, which occupies an area of 1.570 mm^2^. In Figure 11a, the measured small-signal gain (S21) of the power amplifier (PA) ranges from 13.697 dB to 14.478 dB, exhibiting a flatness of 0.781 dB across a frequency range of 6 GHz to 19 GHz. This corresponds to a relative bandwidth of 104%. The small-signal gain reaches its maximum of 14.476 dB at 9 GHz, and the 1 dB bandwidth extends over a wide range of 13.9 GHz, spanning from 5.9 GHz to 19.8 GHz. The chip’s input reflection coefficient S11 is −8.617 dB at 6 GHz, and it improves to better than −10 dB in the frequency range from 7.6 GHz to 19.3 GHz.

During large-signal measurements, the signal generator generates a pulse modulated signal with a 10% duty cycle and 100 μs pulse. Figure 11b presents the large-signal performance measurement results. The power amplifier achieves a Pout ranging from 25.55 to 27.15 dBm, and a PAE ranging from 4.92% to 11.60%.

To evaluate this PA with the TFB technique, a comparison with other ultra-wideband amplifiers is presented in Table 3. Compared to the feedback amplifiers in the CMOS and GaAs processes, the proposed power amplifier has better input return loss and higher output power. Compared to ultra-wideband GaN power amplifiers, it has a more compact area. What is particularly important is that a gain flatness of 0.78 dB is achieved using the TFB bandwidth enhancement technique, which is much higher than the values obtained.

## 5. Conclusions

This paper analyzes the principle of on-chip transformer feedback and proposes a design for an ultra-wideband transformer feedback (TFB) power amplifier (PA) based on GaN 0.25 μm process technology. The C-Ku band broadband power amplifier achieves a relative bandwidth of 117%, a small signal gain of 15 dB, and a saturated output power of 27 dBm under a 28 V power supply condition. Furthermore, by employing the TFB technique, the small signal gain flatness is maintained at only 0.78 dB in the 6 to 19 GHz band, which is suitable for the design of ultra-wideband GaN power amplifiers. Compared with other designs, the on-chip transformer feedback broadband PA has a flatter small signal gain and a more compact topology structure.

## Figures and Tables

**Table 1:** Properties comparison of various semiconductor materials.

Property	Si	SiC	GaAs	InP	AlN	GaN
Energy bandgap (eV)	1.12	3.2	1.43	1.34	6.2	3.4
Breakdown field (10^6^ V/cm)	0.3	3.2	0.4	0.6	11.7	3.3
Electron mobility (cm^2^/V·S)	1500	700	8500	4600	300	2000
Saturation velocity (10^5^ m/s)	1.0	2.0	1.2	1.0	2.0	2.5
Thermal conductivity (W/cm·K)	1.31	4.9	0.46	0.77	3.4	1.5

**Table 2:** Technology process parameter comparison for transformer design.

Technology Process	TSMC 0.13 µm RF CMOS	TSMC 0.18 µm RF CMOS	TSMC 0.25 µm RF CMOS	WIN 0.15 µm InGaAs pHEMT	WIN 0.25 µm GaN on SiC
Number of metal layers	8	6	5	2	2
Min. metal layer space (µm)	/	1.5	/	4	6
Top layer metal thickness (µm)	3.3	2.34	0.99	2	4
Top metal to substrate distance (µm)	7.47	8.15	7.47	5.2	1.62
Top metal conductance (S/m)	5.816 × 10^7^	2.464 × 10^7^	2.464 × 10^7^	4.1 × 10^7^	4.1 × 10^7^
Substrate dielectric constant	11.9	11.9	11.9	12.9	9.7

**Table 3:** Comparison with ultra-wideband amplifier design.

Reference	[21]	[32]	[33]	[34]	This Work
Technology Process	65 nm CMOS	0.15 μm GaAs	0.2 μm GaN	0.25 μm GaN	0.25 μm GaN
Matching Topology	Transformer Feedback	RLC Feedback	Distributed	Non-Foster	Transformer Feedback
Frequency (GHz)	25–35	6–18	2–18	6–18	6–19
S21 (dB)	10	17.4	18	15	15
Gain Flatness (dB)	3	2	3	5.6	0.78
Input Return (dB)	−5	−8	−14	NA	−10
Psat (dBm)	14.75	19.2	29	35.7–37.5	25.55–27.15
PAEsat (%)	40–46.4	13–21.7	5–15	13–21	4.92–11.6
Chip Area (mm^2^)	0.19	0.982	8	8.77	1.571

## Data Availability

The original contributions presented in the study are included in the article, further inquiries can be directed to the corresponding author.
